# Destination of apyrene sperm following migration from the bursa copulatrix in the monandrous swallowtail butterfly *Byasa alcinous*

**DOI:** 10.1038/s41598-020-77683-x

**Published:** 2020-12-01

**Authors:** Tatsuro Konagaya, Naoto Idogawa, Mamoru Watanabe

**Affiliations:** 1 Graduate School of Life and Environmental Sciences, University of Tsukuba, Tsukuba, Japan; 2 Division of Evolutionary Developmental Biology, National Institute for Basic Biology, Okazaki, Japan; 3 College of Biological Sciences, University of Tsukuba, Tsukuba, Japan; 4 Graduate School of Agriculture, Kyoto University, Kyoto, Japan

**Keywords:** Behavioural ecology, Entomology

## Abstract

Most male lepidopterans produce fertile eupyrene sperm and non-fertile apyrene sperm, both of which are transferred to the female in a spermatophore during mating. Apyrene sperm outnumbers eupyrene sperm and both sperm types migrate from the bursa copulatrix to the spermatheca after mating. While eupyrene sperm are maintained in the spermatheca until oviposition, the number of apyrene sperm decreases with time. It is unclear whether apyrene sperm disappear from all sperm storage organs in females because both sperm types are often observed in the spermathecal gland. To investigate this, the numbers of both sperm types were estimated in the spermatheca and spermathecal gland of female *Byasa alcinous* (a monandrous butterfly) 6, 12, 48, 96, and 192 h after mating terminated. Apyrene sperm arrived in the spermatheca earlier than eupyrene sperm; however, some eupyrene and apyrene sperm migrated to the spermathecal gland from the spermatheca at almost the same time. The number of apyrene sperm reached a peak 12 h after the termination of mating and then decreased with time in both the spermatheca and spermathecal gland. Our results suggest that the role of apyrene sperm might be completed early after arriving in the spermatheca of *B. alcinous*.

## Introduction

Sperm polymorphism has been reported in several animal groups including both invertebrates and vertebrates^1^. Males of species with sperm polymorphism generally produce fertile eusperm and non-fertile parasperm^2^; in some species the non-fertile sperm outnumbers the fertile sperm^3^. The role of parasperm production, and the evolutionary scenario that led to its emergence, remains unclear, especially in animals with internal fertilization. In most species of Lepidoptera, males produce fertile eupyrene sperm and non-fertile apyrene sperm, which lack a nucleus^4,5^. In general, apyrene sperm is smaller than eupyrene sperm, but it occupies > 85% of all sperm produced by most butterflies and moths^3^.

It is important to note that apyrene sperm is not the product of failed spermatogenesis. The process of spermatogenesis does differ between eupyrene and apyrene sperm^6,7^. Adult males store apyrene spermatozoa and eupyrene sperm bundles in the sperm storage organ, commonly referred to as the duplex of the seminal vesicle. Each eupyrene sperm bundle is a package of 256 eupyrene spermatozoa^8^. The males transfer a spermatophore containing both types of sperm to the bursa copulatrix of females during mating. Thereafter, eupyrene sperm bundles dissociate and both types of spermatozoa start to migrate to the spermatheca^9^.

Many hypotheses have been proposed to explain the role of apyrene sperm. Several empirical studies support the “cheap filler” hypothesis, which states that apyrene sperm fill the spermatheca to reduce the receptivity of females for re-mating^3,10^. A study of the polyandrous green-veined white butterfly, *Pieris napi*, by Cook and Wedell supports this hypothesis^11^: females that store a larger number of apyrene sperm show a lower receptivity for re-mating. However, this hypothesis is more controversial in other species^12–14^. On the other hand, apyrene sperm is necessary for complete dissociations of eupyrene sperm bundles in *Bombyx mori*^9^. This may be one reason for the sterility and failure of sperm migration in *B. mori* males that produce abnormal apyrene sperm^7,15^. Another hypothesis is that apyrene sperm intrinsically aids eupyrene sperm migration^16^, and another is that apyrene sperm may carry nutrients to the spermatheca that are used by eupyrene sperm or females^17^. These hypotheses are not mutually exclusive; thus, apyrene sperm could have multiple functions^2^.

The dynamics of sperm numbers in female reproductive organs may provide insights into the role of apyrene sperm. Comparisons of such sperm numbers in monandrous and polyandrous species perhaps highlight the competitive function of apyrene sperm in sperm competition. In addition, declines in apyrene sperm might indicate its functional termination^18^. Sperm dynamics in the spermatheca have previously been reported in many polyandrous and a few monandrous species. Apyrene sperm is known to arrive in the spermatheca earlier than eupyrene sperm in both polyandrous^19^ and monandrous species^13^, and the migration of both sperm types ends approximately 1–2 days after mating^20^. While the eupyrene sperm is maintained in the spermatheca until oviposition, the number of apyrene sperm decreases with time^21^. This decline suggests that apyrene spermatozoa may complete their function soon after sperm migration. However, the current data is insufficient to conclude that apyrene sperm disappear from all of the females’ sperm storage organs. Indeed, a considerable number of apyrene sperm often remain in the duct within the spermathecal gland connected with the spermatheca^22^. Although the spermathecal gland is regarded as a secretory organ^23^, it might also function as an additional sperm storage organ. Therefore, to determine the fate of apyrene sperm following migration in females, the sperm dynamics in the spermathecal gland should also be investigated.

**Figure 1:**
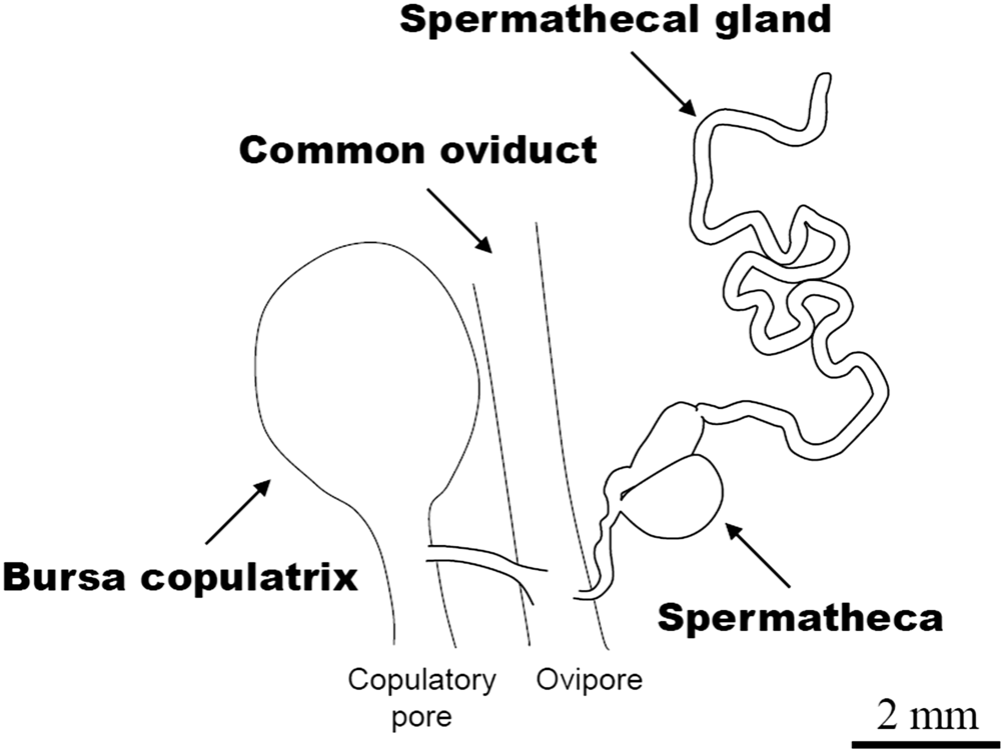
A schematic model of the spermatheca and spermathecal gland of *Byasa alcinous*. Eupyrene and apyrene spermatozoa migrate from the bursa copulatrix to the spermatheca via the common oviduct. A single spermathecal gland is connected with the spermatheca in *B. alcinous*. The length of the spermathecal gland varies among species, and the spermathecal gland is branched in some species^22^.

*Byasa alcinous* is a monandrous swallowtail butterfly for which the mating system is well studied. Typically, females mate only immediately after eclosion for two reasons^24^: (1) the males make a conspicuous mating plug that physically prevents the re-mating of females^25^ and (2) the females seem to have low propensity to re-mating. In fact, females that are forced to mate multiple times suffer reduced longevity and low reproductive output^26^. In such a mating system, apyrene sperm seems not to function as a “cheap filler” to reduce female receptivity for re-mating. However, apyrene sperm occupies 90% of all sperm in *B. alcinous* ejaculates, and the decline in apyrene sperm from the spermatheca has been reported in a spring generation that overwintered in the pupal stage^13^. In the present study, we investigated the dynamics of eupyrene and apyrene sperm in the spermatheca and spermathecal gland of a direct-developing summer generation of *B. alcinous* (Fig. 1).

## Results

Thirty-nine females were dissected. The average forewing length of these *B. alcinous* was 52.1 ± 2.7 mm (mean ± SD). Dissections of the spermatheca and spermathecal gland succeeded for 34 and 36 females, respectively. The length of spermathecal gland was 14.50 ± 2.93 mm (mean ± SD). Two models to explain the length of the spermathecal gland were compared: the null model (BIC = 170.681, df = 32) had a lower BIC value than an alternative model that included forewing length as an explanatory variable (BIC = 173.075, df = 31), indicating that the length of spermathecal gland did not depend on forewing length.

**Figure 2:**
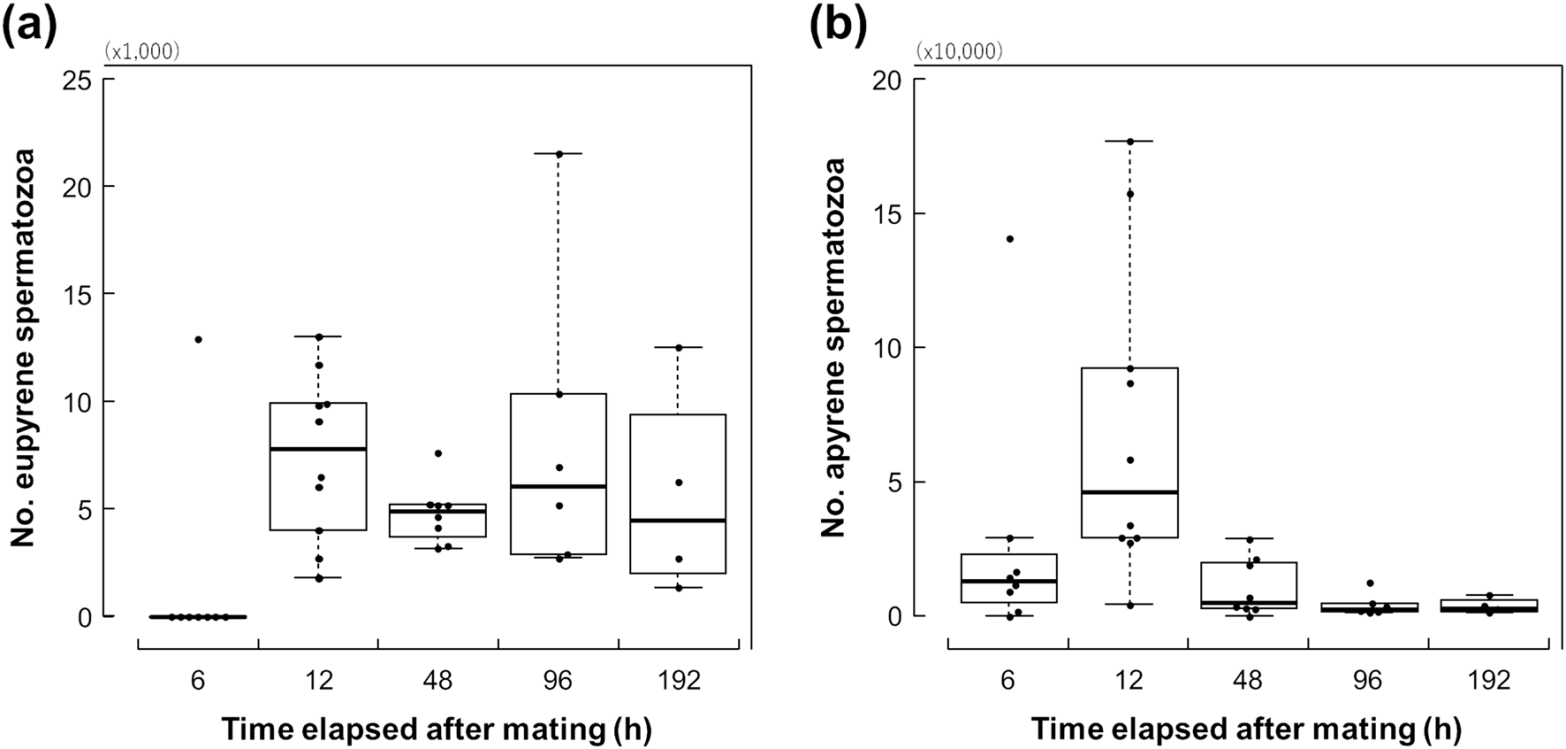
Changes in the number of eupyrene (**a**) and apyrene (**b**) spermatozoa in the spermatheca over time following the termination of mating in *Byasa alcinous*. The boxes represent the median and the interquartile range, and the whiskers indicate maximum and minimum points in 1.5 times interquartile ranges.

We found that apyrene sperm arrived in the spermatheca earlier than eupyrene sperm (Fig. 2). Apyrene spermatozoa appeared in the spermatheca 6 h after the termination of mating in 7 of 8 females examined. In contrast, 6 h after the termination of mating, eupyrene spermatozoa were observed in the spermatheca of only 1 of 8 females.

Apyrene sperm but not eupyrene sperm declined in the spermatheca from 12 h after the termination of mating (Fig. 2). The number of apyrene spermatozoa peaked 12 h after mating terminated (median number = 46,350) and then decreased. A model to explain the number of apyrene spermatozoa that included the time elapsed as an explanatory variable (BIC = 616.448, df = 26) was supported rather than the null model (BIC = 631.365, df = 27). On the other hand, the number of eupyrene spermatozoa was potentially saturated at 12 h after the termination of mating; this number was maintained until at least 192 h after mating ended. The null model (BIC = 543.466, df = 27) to explain the number of eupyrene spermatozoa showed a lower BIC value than the alternative model that included the time elapsed as an explanatory variable (BIC = 546.719, df = 26). For each of the recorded time points after the termination of mating, i.e., 12, 48, 96, and 192 h, the median number of eupyrene spermatozoa was 7790, 4925, 6064, and 4496, respectively.

**Figure 3:**
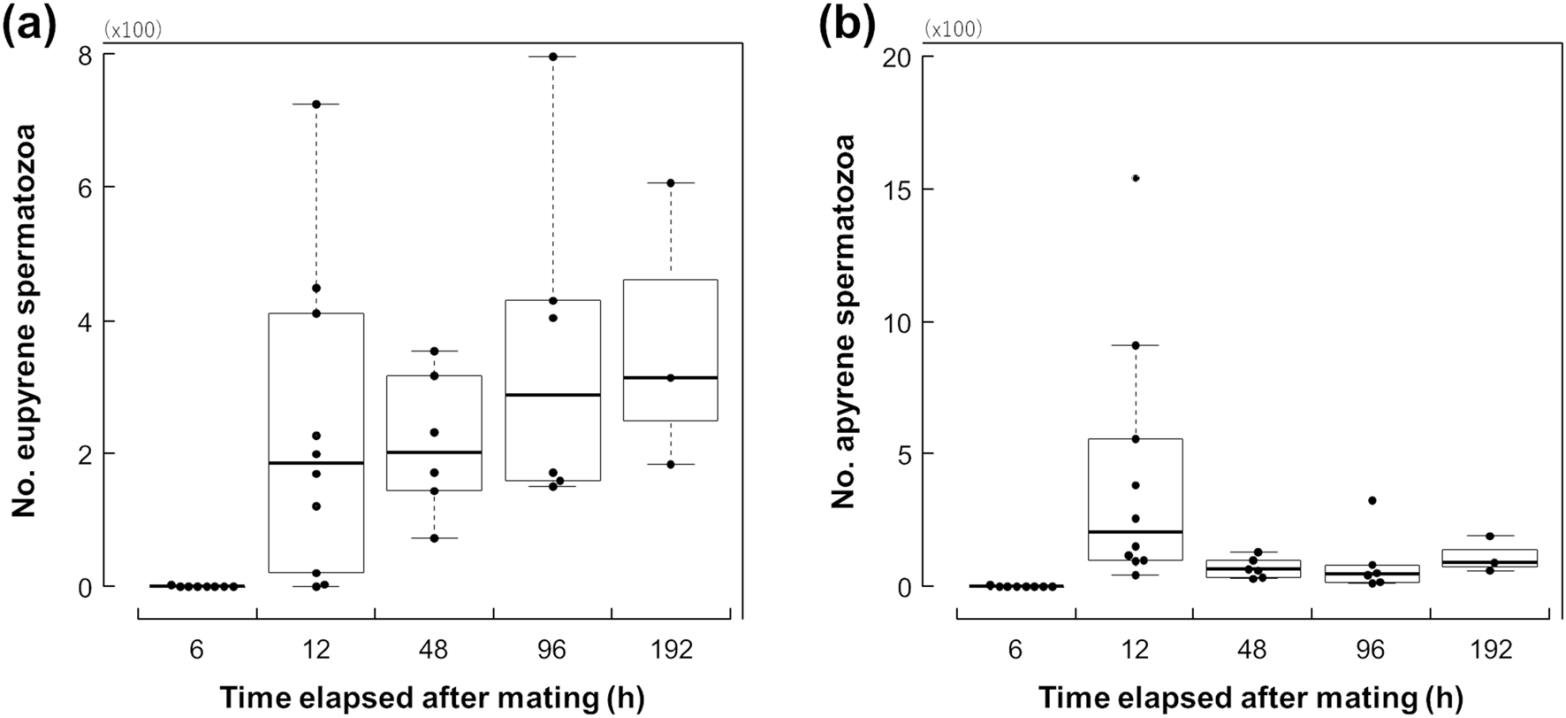
Changes in the number of eupyrene (**a**) and apyrene (**b**) spermatozoa in the spermathecal gland over time following the termination of mating in *Byasa alcinous*. The boxes represent the median and the interquartile range, and the whiskers indicate maximum and minimum points in 1.5 times interquartile ranges.

Early sperm dynamics in the spermathecal gland differed to those in the spermatheca. Few eupyrene and apyrene spermatozoa were observed in the spermathecal gland 6 h after mating ended (Fig. 3). However, both types of spermatozoa appeared in the spermathecal gland 12 h after the termination of mating. In the spermathecal gland, apyrene sperm did not appear to arrive earlier than eupyrene sperm.

An apyrene sperm-specific decline was also observed in the spermathecal gland (Fig. 3). The median number of apyrene spermatozoa was 207 at 12 h after the termination of mating; this number then decreased as time elapsed. The model that explained the number of apyrene spermatozoa with time elapsed (BIC = 323.362, df = 23) had a lower BIC value than the null model (BIC = 325.730, df = 24). In contrast, the number of eupyrene spermatozoa was maintained from 12 h until at least 192 h after mating ended. The null model (BIC = 337.141, df = 24) to explain the number of eupyrene spermatozoa in the spermathecal gland had a lower BIC value than the model containing time elapsed as an explanatory variable (BIC = 339.439, df = 23). For each of the recorded time points, 12, 48, 96, and 192 h after the termination of mating, the median number of eupyrene spermatozoa in the spermathecal gland was 185.5, 202, 288, and 315, respectively.

**Figure 4:**
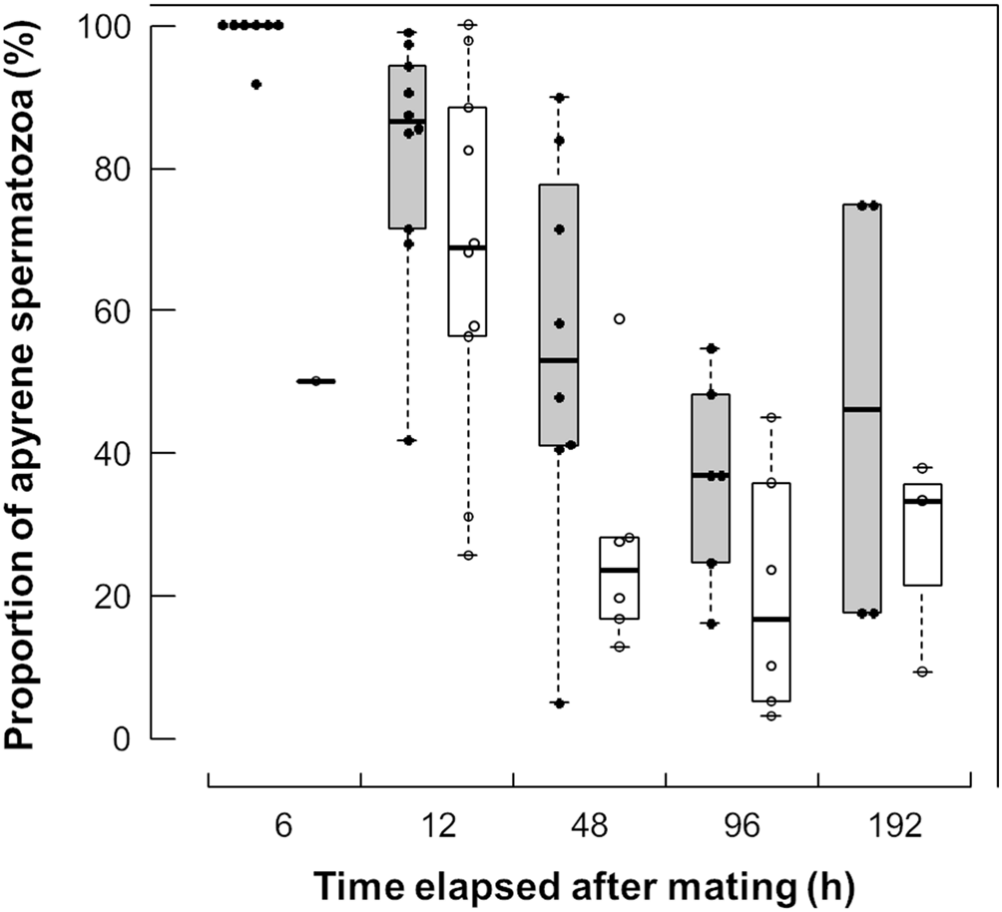
Changes in the proportions of apyrene spermatozoa to all spermatozoa in the spermatheca and the spermathecal gland in *Byasa alcinous*. The boxes represent the median and the interquartile range, and the whiskers indicate maximum and minimum points in 1.5 times interquartile ranges. Closed circles and grey boxes represent the data in the spermatheca. Open circles and white boxes represent the data in the spermathecal gland.

The proportions of apyrene spermatozoa to all spermatozoa were differed between the spermatheca and the spermathecal gland and decreased with time after mating in both organs (Fig. 4). The lowest BIC was obtained from a model including time after mating and female organs as explanatory variables (Table 1). The median proportions of apyrene spermatozoa were always lower in the spermathecal gland than the spermatheca. The appropriate model did not include the interaction between time after mating and female organs as explanatory variables, indicating that the differences for the proportion of apyrene spermatozoa between the spermatheca and the spermathecal gland did not depend on time after mating.

**Table 1:** Results of model selection in analysis of the proportions of apyrene spermatozoa to all spermatozoa within the spermatheca and the spermathecal gland of *Byasa alcinous.*

Model	BIC	ΔBIC
Organs + time after mating	3426.8	0
Organs × time after mating	3428.9	2.1
Organs	3434.2	7.4
Time after mating	5093.7	1666.9
1	5102.2	1675.4

Bayesian information criteria (BIC) values were obtained by generalized linear mixed models (GLMMs) with a binomial distribution. Models including organs as explanatory variables assume the proportions of apyrene sperm differ between the spermatheca and the spermathecal gland. Female ID was included in the models as a random factor.

**Figure 5:**
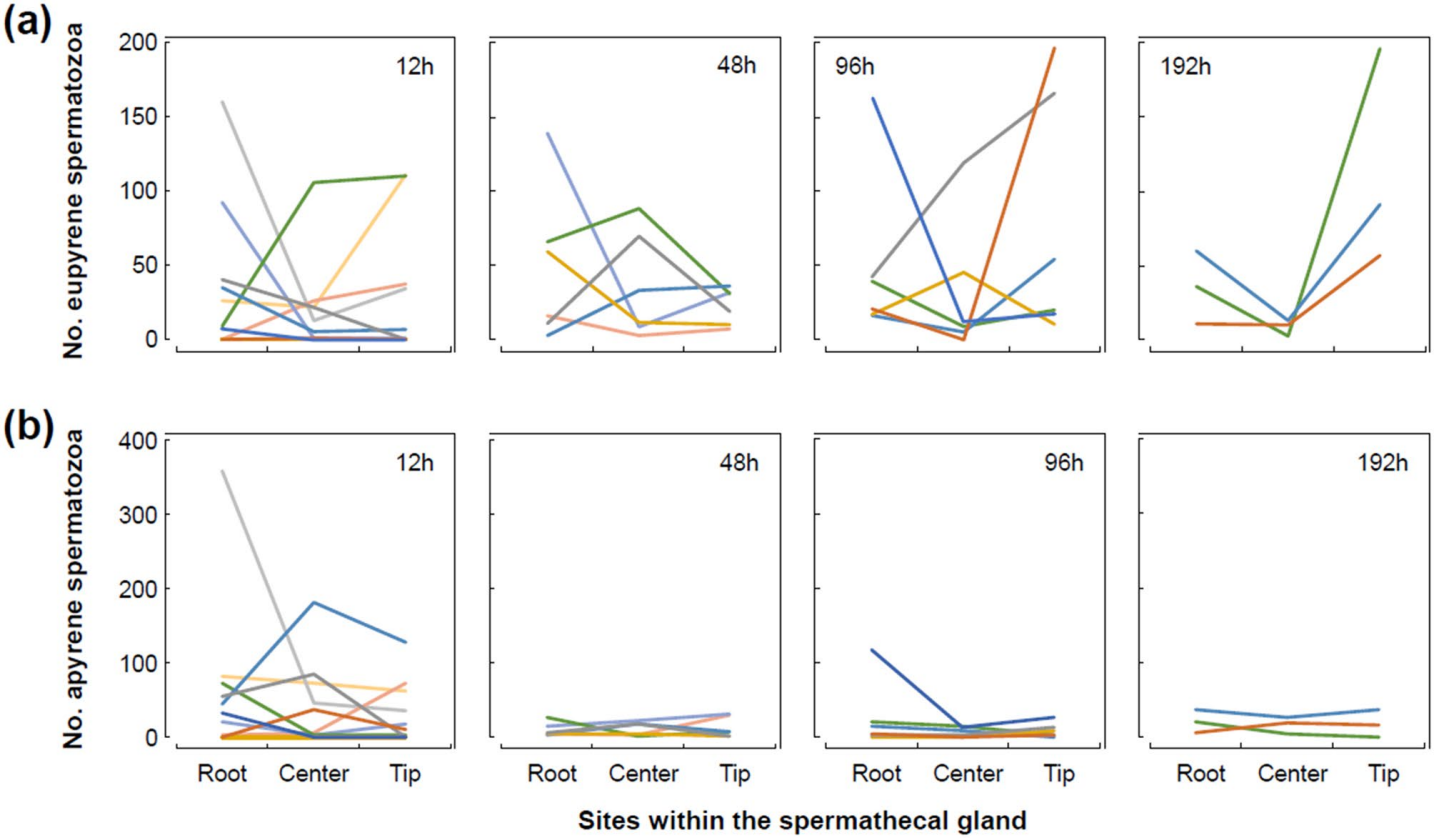
The number of eupyrene (**a**) and apyrene spermatozoa (**b**) in the root, center, and tip of the spermathecal gland at 12, 48, 96 and 192 h after the termination of mating in *Byasa alcinous*. Each line represents the data of each female.

Local existence was rarely observed for both types of sperm in the spermathecal gland (Fig. 5). The lowest BIC was obtained from a model that assumed no differences existed in the number of spermatozoa among each part of the spermathecal gland for all data sets, with the exception of eupyrene sperm at 192 h after the termination of mating (Tables 2, 3). For eupyrene sperm at 192 h, the largest number of spermatozoa was recorded in the tip of the spermathecal gland and this number decreased from the root to the center of the spermathecal gland. The model with the lowest BIC assumed that the number of spermatozoa differed among the parts of the spermathecal gland for eupyrene sperm at 192 h post-mating.

**Table 2:** Results of model selection in analysis of the local existence of eupyrene spermatozoa within the spermathecal gland of *Byasa alcinous*.

Time after mating	Model	BIC	ΔBIC
12 h	(Root, Center, Tip)	242.707	0
	(Root, Tip) (Center)	245.611	2.904
	(Center, Tip) (Root)	245.803	3.096
	(Root, Center,) (Tip)	246.101	3.394
	(Root) (Center) (Tip)	248.952	6.245
48 h	(Root, Center, Tip)	173.757	0
	(Root, Center,) (Tip)	174.937	1.180
	(Center, Tip) (Root)	175.349	1.592
	(Root, Tip) (Center)	176.647	2.890
	(Root) (Center) (Tip)	177.469	3.712
96 h	(Root, Center, Tip)	187.046	0
	(Root, Tip) (Center)	188.546	1.500
	(Root, Center,) (Tip)	188.564	1.518
	(Center, Tip) (Root)	189.909	2.863
	(Root) (Center) (Tip)	190.969	3.923
192 h	(Root) (Center) (Tip)	87.298	0
	(Root, Tip) (Center)	90.036	2.738
	(Root, Center,) (Tip)	91.242	3.944
	(Root, Center, Tip)	96.182	8.884
	(Center, Tip) (Root)	97.865	10.567

The words in parentheses represent parts of the spermathecal gland. In each model, the parts within the same parenthesis were treated as a single category. Bayesian information criteria (BIC) values were obtained using generalized linear mixed models (GLMMs) with a negative binomial distribution. Female ID was included in the models as a random factor.

**Table 3:** Results of model selection in analysis of the local existence of apyrene spermatozoa within the spermathecal gland of *Byasa alcinous*.

Time after mating	Model	BIC	ΔBIC
12 h	(Root, Center, Tip)	285.084	0
	(Center, Tip) (Root)	287.572	2.488
	(Root, Center,) (Tip)	287.834	2.750
	(Root, Tip) (Center)	288.431	3.347
	(Root) (Center) (Tip)	290.829	5.745
48 h	(Root, Center, Tip)	133.076	0
	(Root, Center,) (Tip)	135.721	2.645
	(Center, Tip) (Root)	135.779	2.703
	(Root, Tip) (Center)	135.960	2.884
	(Root) (Center) (Tip)	138.566	5.490
96 h	(Root, Center, Tip)	132.510	0
	(Center, Tip) (Root)	133.567	1.057
	(Root, Tip) (Center)	134.736	2.226
	(Root, Center,) (Tip)	134.986	2.476
	(Root) (Center) (Tip)	136.452	3.942
192 h	(Root, Center, Tip)	77.039	0
	(Center, Tip) (Root)	79.062	2.023
	(Root, Tip) (Center)	79.171	2.132
	(Root, Center,) (Tip)	79.212	2.173
	(Root) (Center) (Tip)	81.257	4.218

The words in parentheses represent parts of the spermathecal gland. In each model, the parts within the same parenthesis were treated as a single category. Bayesian information criteria (BIC) values were obtained using generalized linear mixed models (GLMMs) with a negative binomial distribution. Female ID was included in the models as a random factor.

The length of the spermathecal gland did not explain the number of spermatozoa that reached or were stored in the spermatheca. When females from 12 to 192 h post-mating were pooled, the model for explaining the number of eupyrene spermatozoa in the spermatheca according to the length of spermathecal gland (BIC = 450.566, df = 21) had a higher BIC value than the null model (BIC = 447.461, df = 22). In addition, the null model also explained the number of apyrene spermatozoa in the spermatheca 12 h after mating (BIC = 247.009, df = 9) better than the model that included the length of the spermathecal gland as an explanatory variable (BIC = 249.296, df = 8).

## Discussion

Apyrene sperm have been suggested to play competitive roles in polyandrous species^11,19^. For example, when these sperm function as the “cheap filler,” the large number of apyrene spermatozoa in the spermatheca is important for reducing female receptivity for re-mating^10^. However, rapid declines in apyrene spermatozoa from the spermatheca have been quantitatively demonstrated in polyandrous species such as *Pseudaletia separate*^20^, *Papilio xuthus*^19^, and *Eurema mandarina*^12^. A similarly rapid decline in apyrene spermatozoa was also shown quantitatively in the spring generation of the monandrous butterfly *B. alcinous*^13^. The present study confirmed that apyrene spermatozoa specifically declined in the spermatheca of a summer generation of *B. alcinous.* Thus, the decline of apyrene spermatozoa may be a common feature of the sperm dynamics of lepidopteran insects irrespective of their mating system.

In a study of the polyandrous swallowtail butterfly species *P. xuthus*, Watanabe et al. demonstrated that apyrene spermatozoa appeared in the spermatheca earlier than eupyrene spermatozoa^19^, and they suggested that apyrene spermatozoa push the eupyrene spermatozoa of competing males too far from the opening of the spermatheca when females mate on multiple occasions. However, this theory is unlikely to apply to monandrous species such as *B. alcinous*, in which the earlier migration of apyrene spermatozoa to spermatheca has also been shown in a spring generation^13^. In the present study, we also found that apyrene spermatozoa migrated to the spermatheca earlier than eupyrene spermatozoa in a summer generation of *B. alcinous*. Because *B. alcinous* females have low propensity to re-mate^26^, the earlier migration of apyrene spermatozoa is apparently not associated with sperm competition.

Our results also showed that some eupyrene and apyrene spermatozoa moved into the spermathecal gland from the spermatheca, and that apyrene sperm specifically decreased as time elapsed. These results are inconsistent with those of a previous study of *P. xuthus*, in which eupyrene sperm was rare in the spermathecal gland and the number of apyrene sperm (ca. 400) did not decrease with time after mating^27^. This discrepancy might reflect differences between polyandrous and monandrous mating systems; however, in this previous study, female *P. xuthu*s were fed only water after mating despite the nutritional status of females possibly being a factor that affects sperm storage in insects^28^.

In the most extreme case observed in the present study, a female *B. alcinous* contained approximately 800 eupyrene spermatozoa in its spermathecal gland. This number is still lower than the observed number of eupyrene sperm in the spermatheca of this species. However, it is comparable to the number of eupyrene spermatozoa in the spermatheca of *Plodia interpunctella* (200 recorded)^14^ as well as *Pieris napi*^11^, *Papilio xuthus*^19^, and *E. mandarina*^12^ (all 1000). Although we have no data on the fertility of eupyrene sperm in the spermathecal gland, our results suggest that lepidopteran females may store much more sperm than we previously expected.

The delay of sperm migration into the spermathecal gland is notable when estimating the functions of the spermathecal gland of butterflies. Although a considerable number of apyrene spermatozoa were already in the spermatheca of most females 6 h after mating, migration into the spermathecal gland was first detected 12 h after mating. Thus, the spermathecal gland may open once the eupyrene sperm arrives at this time. The spermathecal gland may also be associated with the decomposition of apyrene sperm^22^ because the numbers decreased as time elapsed. This hypothesis could explain the relatively lower proportion of apyrene spermatozoa in the spermathecal gland than in the spermatheca. Alternatively, the delay in sperm migration into the spermathecal gland could relate to the production of spermathecal gland secretions that maintain eupyrene spermatozoa^20,23,29^; in this scenario, the spermathecal gland might open to transfer the secretions to the spermatheca around 12 h after mating. However, the number of eupyrene sperm stored in the spermatheca seemed not to depend on the length of spermathecal gland.

Seasonal variations are inconspicuous in the number of eupyrene spermatozoa stored in the spermatheca. The median and the mean numbers of eupyrene spermatozoa in the spermatheca 12–196 h after mating are approximately 5700 and 5600, respectively for the spring generation of *B. alcinous*^13^. These are comparable to 5200 and 6600 in the present study. In *Polygonia c-aureum* showing adult diapause, non-diapause males have larger numbers of cysts in their testis than diapause-males probably because of their large body size^30^. For *B. alcinous* that overwinter as pupae, adults of the summer generation also show larger body size than that of the spring generation overwintered^31^. However, the ability to produce sperm cannot be compared between the spring and summer generations in *B. alcinous,* because the present study did not examine the number of spermatozoa in the testis or the duplex.

The present study suggests that the final destination of apyrene sperm following migration is the spermathecal gland because (1) some apyrene spermatozoa migrated to the spermathecal gland from the bursa copulatrix via the spermatheca and (2) apyrene sperm declined in the spermathecal gland (which is essentially a cul-de-sac). The decline of apyrene sperm may coincide with their decreased motility in the spermatheca^13^. Indeed, the apyrene sperm of *B. alcinous* might complete their role soon after arriving in the spermatheca. Thus, the present results are consistent with the hypothesis that apyrene spermatozoa help eupyrene spermatozoa to migrate to the spermatheca from the bursa copulatrix^16^. However, sperm dynamics in the spermathecal gland seem to differ between monandrous *B. alcinous* and polyandrous *P. xuthus*^27^, which suggests that apyrene sperm might play a competitive role in polyandrous species. Further studies of the sperm in the spermathecal gland will help to determine the competitive roles of apyrene sperm in polyandrous species.

## Methods

Pupae of *B. alcinous* were collected in Tsukuba City, Ibaraki Pref., Japan in the summer of 2014. When adults emerged, they were sexed and their forewing length was measured using a digital venire caliper (accuracy of 0.1 mm). Males and females were maintained in separate net cages (40 × 40 × 50 cm). The adult butterflies were fed 20% sucrose solutions for 5 min every day.

Two-day-old virgin females were mated with 2–8-day-old virgin males by the hand-pairing method. The mated females were not allowed to oviposit; they were dissected at 6, 12, 48, 96, and 192 h after the termination of mating. The spermatheca including the spermathecal gland was removed from females and placed in 10 μl of insect saline solution (128.4 mM NaCl, 4.7 mM KCl, and 1.9 mM CaCl_2_) on a glass slide. The spermathecal gland was then removed onto another slide. The spermathecal gland of *B. alcinous* is a simple blind duct without any branches.

In the drop of insect saline solution, the spermatheca was opened and the ejaculate was diluted with 2 ml of the insect saline solution or 6 ml when the sperm density seemed to be high. After gentle homogenization, six 10-μl drops of diluted ejaculate were dried on a slide for each sample. The slide was washed for 5 s in distilled water and then allowed to dry. The numbers of eupyrene and apyrene spermatozoa on a slide were counted under a stereoscopic microscope (100×). Both types of sperm are easily distinguished by their morphology^13^. The number of spermatozoa in the spermatheca was calculated by multiplying the mean number of sperm in the suspension by its dilution factor.

The length of the spermathecal gland was obtained using a computer-based image-processing system (accuracy of 0.01 mm; AZ100M with Nikon NIS-Elements ver.3.2; Nikon, Japan). For each sample image, a line was drawn along the center of the spermathecal gland and the length of the line was measured. Following this measurement, the tip, center, and root of the spermathecal gland were dissected out by approximately 2 mm, respectively. Each individual part was dissected out in a drop of the insect saline solution on a slide. After drying, the slide was washed for 5 s in distilled water and dried. Both types of spermatozoa in each part of the spermathecal gland were counted and pooled for each female. The number of spermatozoa in a spermathecal gland was estimated by multiplying a sixth of the total number of spermatozoa counted (the number of spermatozoa in 1 mm of the spermathecal gland) by the total length of the spermathecal gland (mm).

Statistical analyses were performed in R version 3.6.2^32^. Linear regression analysis was used to assess the relationship between forewing length and spermathecal gland length. A generalized linear model (GLM) was used to analyze changes in the number of spermatozoa in the spermatheca and the spermathecal gland from 12 to 192 h after mating. In these analyses, the time elapsed after the termination of mating was used as an explanatory variable and a negative binomial distribution was assumed. The glm.nb function in the MASS package was used to conduct these analyses. Relationships between the number of spermatozoa in reproductive organs and the spermathecal length were also analyzed by GLMs assuming a negative binomial distribution. Bayesian information criteria (BIC) were used to select models according to the importance of explanatory variables. BIC is an appropriate criterion for model selection in controlled experiments^33^. In each analysis, the BIC value of the null model, which contained no explanatory variables, was compared with an alternative model containing an explanatory variable. The model with the lowest BIC value was favored. We considered one model to be superior when the difference between the BIC values of the best model and other models was > 2^34^.

The proportion of apyrene spermatozoa to all spermatozoa was analyzed by a generalized linear mixed model (GLMM) assuming a binomial distribution. The glmer function in the lme4 package was used to conduct these analyses. Female ID was incorporated as a random factor. Female organs that the proportions of apyrene spermatozoa were investigated, and time after mating were treated as potential explanatory variables. The BIC values were compared among models fitted.

The local existence of each type of spermatozoa in the spermathecal gland was analyzed by a GLMM assuming a negative binomial distribution. The glmer.nb function in the lme4 package was used to conduct these analyses. Female ID was incorporated as a random factor. Data for 12, 48, 96, and 192 h after the termination of mating were separately analyzed. Five models were fitted. First, the null model assumed no differences among each part of the spermathecal gland: (*(Root, Center, Tip)*). Second, a model was considered in which the number of spermatozoa in each part of the spermathecal gland differed from each other part within an individual: (*(Root) (Center) (Tip)*). The third, fourth, and fifth models involved a part in the spermathecal gland containing a different number of spermatozoa from other parts: (*(Root, Center) (Tip)*), (*(Root, Tip) (Center)*), and (*(Root) (Center, Tip)*), respectively. Finally, the BIC values of these models were compared.
